# Development of a Simple and Validated LC–MS/MS Method for Quantitative Determination of Ketotifen in Beagle Dog Plasma and Its Application to Bioequivalence Study of Ketotifen Syrup Dosage Form

**DOI:** 10.3390/molecules29184505

**Published:** 2024-09-23

**Authors:** Eunseo Song, Wang-Seob Shim, Doowon Choi, Yuna Song, Hyeong Geun Jo, Soobok Lee, Suk Han Jung, Yeo Jin Choi, Kyung-Tae Lee

**Affiliations:** 1 Department of Biomedical and Pharmaceutical Science, Graduated School, Kyung Hee University, Seoul 02447, Republic of Korea; sssk2303@khu.ac.kr (E.S.); atnoon@khu.ac.kr (D.C.); songyu0819@khu.ac.kr (Y.S.); 2 Kyung Hee Drug Analysis Center, College of Pharmacy, Kyung Hee University, Seoul 02447, Republic of Korea; wsshimm@khu.ac.kr; 3 Department of Pharmacy, College of Pharmacy, Kyung Hee University, Seoul 02447, Republic of Korea; 4 Daewon Pharm. Co., Ltd., 386 Cheonhodae-ro, Gwangjin-gu, Seoul 04808, Republic of Korea; jhgeun@daewonpharm.com (H.G.J.); sblee@daewonpharm.com (S.L.); kshjung@daewonpharm.com (S.H.J.); 5 Department of Regulatory Science, Graduated School, Kyung Hee University, Seoul 02447, Republic of Korea; 6 Department of Fundamental Pharmaceutical Science, Graduated School, Kyung Hee University, Seoul 02447, Republic of Korea

**Keywords:** ketotifen, LC–MS/MS, Beagle dog plasma, syrup, bioanalytical method validation, bioequivalence

## Abstract

A highly accurate, precise, and simple liquid chromatography-tandem mass spectrometry (LC–MS/MS) method for ketotifen (KTF) estimation from Beagle dog plasma was developed and validated, with ketotifen-d3 (KTF-d3) as the internal standard (IS). KTF and IS were detected on an API 4000 mass spectrometer in multiple reaction monitoring (MRM) mode in electrospray ionization (ESI) positive ionization mode. The transitions were monitored at *m/z* 310.2 → 96.0 for KTF and *m/z* 313.2 → 99.1 for IS. KTF and IS were extracted from plasma using liquid-liquid extraction with methyl tertiary-butyl ether and then analyzed for 3 min with extracted samples (7 µL) into the LC–MS/MS system. Analytes were separated on a Luna^®^ Hilic column (50 × 2.0 mm i.d., 3 μm) using the Nexera X2 HPLC. The mobile phase A consisted of 10 mmol/L ammonium formate (pH 3.0), while mobile phase B consisted of 0.05% formic acid in acetonitrile. The ratio of mobile phase was 5:95 (*v*/*v*) at a flow rate of 0.2 mL/min. The method has been thoroughly validated in accordance with the bioanalytical method validation guidelines established by the Ministry of Food and Drug Safety in Korea and the U.S. Food and Drug Administration, addressing selectivity, lower limit of quantification, linearity, carryover, precision, accuracy, recovery, matrix effect, and stability. The developed LC–MS/MS method was effectively utilized for the bioequivalence assessment of ketotifen in Beagle dog plasma following the oral administration of ketotifen syrup.

## 1. Introduction

Ketotifen, also known as 4,9-dihydro-4-(1-methyl-4-piperidinylidene)-10H-benzo [4,5]cyclohepta[1,2-b]thiophen-10-one is commonly utilized as an anti-allergic and antianaphylactic medication for both adults and children [1,2]. It is widely used in the treatment of allergic disorders, such as allergic conjunctivitis, chronic rhinitis, urticaria, and asthma [3]. This drug acts as a selective, noncompetitive inhibitor of histamine H1 receptors and a mast cell stabilizer, preventing the discharge of inflammatory substances from mast cells [2,4,5,6].

Ketotifen is largely absorbed into the bloodstream after being taken orally. Nevertheless, its ability to reach the bloodstream is limited to about 50% due to the hepatic first-pass metabolism [3,7,8]. The oral administration of this drug leads to its absorption, with the highest concentration in the plasma being reached between 2 and 4 h after administration in standard dosage forms [3]. KTF’s plasma concentrations were found to be extremely low, ranging from pg/mL to low ng/mL, due to its low oral dose (2 mg/day) and extensive first-pass metabolism [6,7]. Owing to these characteristics, it is crucial to devise sensitive analytical methods that can identify low concentrations of KTF in plasma samples. There are numerous studies on the pharmacokinetics of oral administration in tablet and capsule forms; however, there is a lack of research on the pharmacokinetics of syrup formulations [9,10]. Syrup formulations generally exhibit pharmacokinetic significance as they are absorbed rapidly, reaching peak concentration (*C*_max_) quickly and showing higher concentrations. According to a previous study, when comparing KTF capsule and syrup formulations, the *C*_max_ was higher in the syrup formulation (421 ± 206 pg/mL) than in the capsule formulation (281 ± 81 pg/mL). Additionally, the *T*_max_ was shorter in the syrup formulation at 2.9 ± 1.9 h compared to 3.9 ± 2.2 h in the capsule formulation, indicating a faster trend in syrup [11].

Previous studies have utilized GC–MS to detect the concentration of KTF in human plasma [12,13,14]. However, this method requires a large volume of plasma (1 mL) and a long run time (>20 min). Various plasma analysis methods using LC–MS/MS or HPLC have been introduced, but some have a high lower limit of quantification (LLOQ) or require large volumes of plasma (400–500 μL of plasma) owing to the low sensitivity of the drug [1,7,10,15]. In addition, there are cases where a small amount of reconstitution solvent is added or the injection volume is increased, but it is preferable to avoid these methods as they can easily lead to contamination of the mass quadrupole [9].

As sugar alcohols have been known to influence drug absorption [16,17], various sugar alcohols are utilized in different ketotifen syrups. Specifically, d-sorbitol is employed in Daewon ketotifen^®^ syrup, while maltitol is used in Zaditen^®^ syrup. Therefore, this study aimed to examine the bioequivalence of two ketotifen syrups, consisting of different sugar alcohols, in Beagle dogs before applying the findings to human models. Considering that the bioequivalence study was conducted in Beagle dogs, the goal was to minimize the blood volume collected from experimental animals. Therefore, developing an analytical method that is sensitive with minimal plasma volume is crucial. Consequently, this study aimed to create a sensitive and rapid analytical method capable of detecting low KTF concentrations in Beagle dogs.

## 2. Results and Discussion

### 2.1. Method Development

#### 2.1.1. Mass Spectrometry

**Figure 1:**
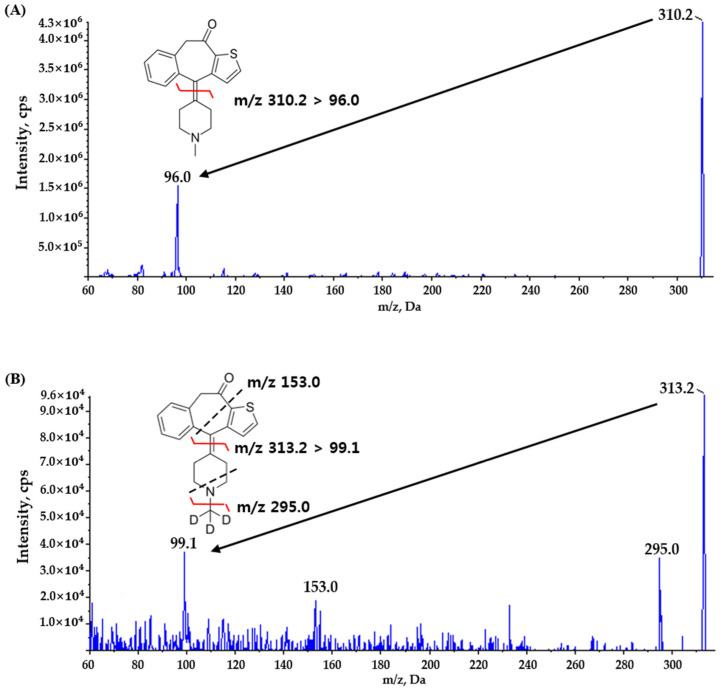
Product ion spectra and the pattern of fragmentation for (**A**) ketotifen and (**B**) ketotifen-d3.

Existing literature did not use structural analogues and stable isotope labels as internal standards, and the reason for using KTF-d3 as IS is that interference between the analyte and IS from the matrix can be reduced. To attain optimal sensitivity and generate ideal fragment ions, the mass spectrometric parameters were adjusted by infusing a solution of KTF (STD) and KTF-d3 (IS) in 100% methanol. The flow rate of 10 μL/min was employed for the syringe pump to deliver the infusion directly to the ionization source. The greatest amount of product ions was obtained in the positive mode through the use of a turbo ion spray ESI interface. Due to the full scan of Q1, KTF was carried out with a fragmentation of *m/z* 310.2 → 96.0 and IS at *m/z* 313.2 → 99.1 product ion in positive mode (Figure 1). This fragmentation of KTF result corresponds to that previously reported [7,9].

#### 2.1.2. Chromatographic Conditions

The optimal chromatographic conditions were established to ensure the efficient and successful separation of KTF and d3-IS from matrix. Various columns, mobile phases, and isocratic ratios were tested to obtain selective peaks for the target analyte and IS under reverse-phase chromatographic conditions. Hydrosphere^®^ C18, Luna^®^ C18, Halo^®^ C18, and Luna^®^ Hilic columns were tested for optimization in our study. While most columns exhibited poor peak shapes or difficulties in separation due to matrix peaks from Beagle dog plasma (Figures S1–S3), the Luna^®^ Hilic (50 × 2.0 mm, 3 μm, Phenomenex) column showed superior separation efficiency and sensitivity when run at a flow rate of 0.2 mL/min. KTF is a polar compound, making it suitable for separation using HILIC (Hydrophilic Interaction Liquid Chromatography) columns, which are effective for separating polar compounds. Using the Hilic column as an alternative to normal-phase, ion-exchange, or ion-pairing chromatography can improve the retention of very polar compounds that are typically poorly retained by conventional reversed-phase LC [18,19]. Finally, the analytes were separated from the plasma matrix. As mentioned in our previous study, initially, we flowed mobile phase A with 10 mmol/L ammonium formate adjusted to pH 3.0, and mobile phase B as 100% acetonitrile in an isocratic mode at a ratio of 5:95 (*v*/*v*) [9]. However, we observed a slight delay in the retention times of KTF and IS. To address this issue, we adjusted the pH of the mobile phase. We lowered the pH by adding formic acid to mobile phase B and confirmed that the most reproducible and stable retention times were achieved with acetonitrile containing 0.05% formic acid. The chromatographic conditions as described above yielded the most favorable peak shapes, separations, sensitivity, and reproducibility.

#### 2.1.3. Plasma Sample Preparation

Different techniques have been documented for extracting KTF from human plasma, including liquid–liquid extraction (LLE), solid-phase extraction (SPE), and protein precipitation (PP) extraction methods, among which the LLE analysis method is the most widely used [1,7,9,10,11,14]. In practice, LLE offers the advantage of obtaining cleaner extracts and excellent selectivity by finely tuning the solvent and pH. As a result, hundreds of LLE extraction samples can be analyzed with good sensitivity using LC–MS/MS without compromising the instrument’s performance [20]. To establish the optimal extraction conditions, experiments were conducted using a mixture of hexane:isopropyl alcohol (99:1, *v*/*v*) and MTBE as extraction solvents [1,9]. The results showed higher recovery rates for KTF with MTBE than for a mixture of hexane/isopropyl alcohol (99:1, *v*/*v*). Next, the reconstitution solvent was tested using 95% acetonitrile, as reported previously [9], but this was found to compromise the pre-treatment stability. Therefore, the mixture of mobile phase B and 95% acetonitrile (5:5, *v*/*v*) was selected as the reconstitution solvent, which showed more stable results. In our study, we achieved optimal recovery rates and high stability of the analyte by using 5 mL MTBE as the extraction solvent, 20 μL of 10 mmol/L sodium hydroxide solution as the pre-treatment buffer, and a mixture of mobile phase B and 95% acetonitrile (5:5, *v*/*v*) as the reconstitution solvent. The reconstructed volume was 300 μL. This approach also facilitates rapid and efficient analysis of numerous plasma samples by requiring only a minute sample volume (7 μL) owing to its exceptional recovery efficiency. In our previous study, we established conditions with a reconstruction volume of 100 μL and an injection volume of 10 μL. In the current study, we established conditions with a greater reconstruction volume and a smaller injection volume. Thus, by using a reconstitution volume of 300 μL, we were able to sufficiently re-dissolve the residue after nitrogen evaporation, and despite the low injection volume, we achieved an S/N ratio of over 10 at the LLOQ.

### 2.2. Method Validation

#### 2.2.1. Selectivity and Lower Limit of Quantitation

**Figure 2:**
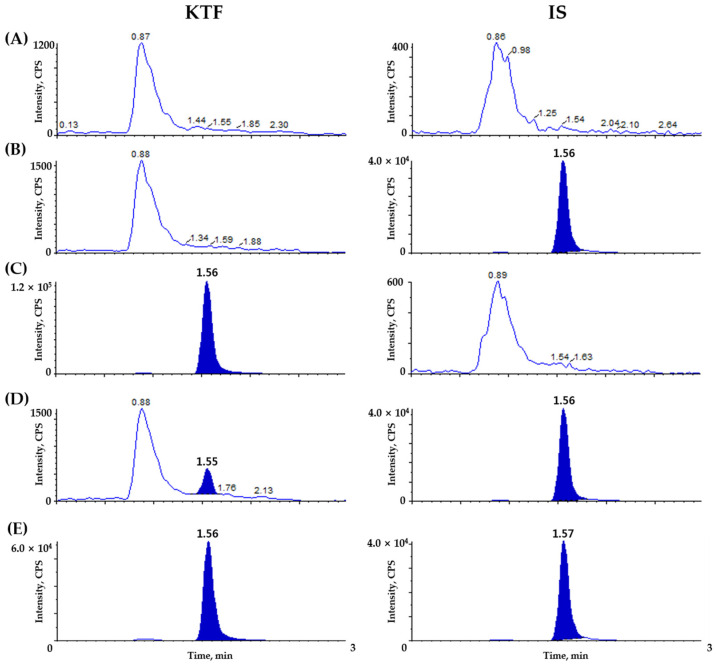
Chromatograms depicting the representation of (**A**) double blank plasma (without ketotifen and IS), (**B**) blank plasma spiked with ketotifen-d3 (IS, 25 ng/mL), (**C**) blank plasma spiked with ketotifen (ULOQ, 5 ng/mL), (**D**) blank plasma spiked with ketotifen (LLOQ, 0.02 ng/mL) and ketotifen-d3 (IS, 25 ng/mL), and (**E**) sample in Beagle dog plasma 1.5 h after oral administration of ketotifen (2 mg/mL/dog) syrup.

To confirm selectivity, we prepared plasma samples from six different origins of Beagle dogs and pooled them. Figure 2 represents blank Beagle dog plasma, blank Beagle dog plasma spiked with KTF-d3 (25 ng/mL, IS), blank Beagle dog plasma spiked with KTF (5 ng/mL), blank Beagle dog plasma spiked with KTF (0.02 ng/mL) and IS (25 ng/mL, IS), and plasma obtained 1.5 h after oral administration of KTF (2 mg/mL/dog) to Beagle dogs followed by spiking with IS (25 ng/mL). In Beagle dog plasma, no interference was detected from endogenous compounds at the elution times corresponding to KTF and IS. Additionally, the determination of the LLOQ (0.02 ng/mL) of KTF was confirmed by achieving a S/N ratio > 10 in plasma samples.

#### 2.2.2. Linearity and Carryover

Calibration plots of KTF exhibited satisfactory linearity within the standard calibration curve range of 0.02–5 ng/mL. The mean slope of the regression equation was determined to be 0.5823 (CV: 3.2%). As indicated in Table 1, all curves demonstrated excellent linearity with *r* values exceeding 0.997, ensuring robustness and reproducibility. Furthermore, no carryover was observed for both KTF and IS at the ULOQ and LLOQ concentrations in Beagle dog plasma.

#### 2.2.3. Precision and Accuracy

The precision and accuracy of KTF were assessed by performing five replicate analyses on concentrations including LLOQ (0.02 ng/mL), LQC (0.06 ng/mL), MQC (0.4 ng/mL), and HQC (4 ng/mL), both for intra-day and inter-day evaluations. The precision of the KTF determination method varied between 2.28% and 6.67% for intra-day measurements and between 3.14% and 7.56% for inter-day measurements. The accuracy of the method for measuring KTF concentrations ranged from 97.56% to 106.31% (Table 2). According to the guidelines set forth by the MFDS and USFDA for bioanalytical applications, all of the results fell within the specified ranges for precision (%) and accuracy (%) [21,22].

#### 2.2.4. Recovery and Matrix Effect

Table 3 represents the extraction recovery and matrix effect of KTF and IS (25 ng/mL) at three QC concentrations (0.06, 0.4, and 4 ng/mL, *n* = 6). To evaluate the recovery of three QC concentrations and IS, the mean peak area values of extracted samples were compared with those of respective aqueous standard solutions directly injected. The average recovery for KTF ranged from 99.71% to 102.30%, with a mean %CV ranging from 5.01% to 10.12%. For IS, the average recovery was 92.40% with a mean %CV of 7.94%. According to the previous reports using a mixture of hexane/isopropyl alcohol (99:1, *v*/*v*) as the extraction solvent, the recovery of KTF was 92.64 ± 2.39%, 85.54 ± 6.83%, and 101.79 ± 3.81% at LQC, MQC, and HQC levels, respectively. Furthermore, in the same study, when oxybutynin chloride was used as the internal standard, the extraction efficiency of the IS was 79.134 ± 6.83%, which was much lower than that of IS, the deuterated analog of KTF, in our research [9]. Thus, we confirmed that the recovery rates were higher (7.58% and 13.27% at analyte and internal standard) in this developed analytical method, and there was no significant difference between the two substances, making it a more effective analytical method.

The method to determine the matrix effect involves comparing the mean peak area values of analyte and IS spiked into extracted blank Beagle dog plasma with those of respective aqueous standard solutions directly injected. The average matrix effect for KTF ranges from 67.22% to 73.93%, with a mean %CV ranging from 6.91% to 12.84%. For IS, the average matrix effect was 71.11% with a mean %CV of 9.58%. Therefore, we confirmed that our developed analytical method showed no mutual interference between endogenous substances within the Beagle dog matrix, KTF, and IS. Furthermore, this confirms the lack of ion enhancement or suppression effects.

#### 2.2.5. Stability

The stability of KTF under various conditions was assessed for stock solutions, working solutions, and Beagle dog plasma in Table 4. Comparing the low and high QC peak areas of KTF from freshly prepared stock solutions with those stored at room temperature for 3 h, the mean % peak area ranged from 97.70–103.54%. Similarly, for freshly prepared working solutions compared with those stored at room temperature for 7 h, the mean % peak area for KTF and IS ranged from 96.20–99.83%. The average stability of KTF in Beagle dog plasma was observed to be 93.33–102.72% when stored at room temperature for 7 h, 96.67–100.93% at 4 °C for 7 h, and 96.33–104.44% at −70 °C for 7 h. Additionally, the stability after three freeze-thaw cycles was found to be 96.25–105.56%, and when samples were kept in an autosampler set at 10 °C after LLE extraction for 30 h, the stability was 88.83–105.52%. Because all deviations fell within the acceptable criteria of ±15% under all test conditions, it was confirmed that KTF was stable in stock solutions, working solutions, and Beagle dog plasma.

#### 2.2.6. Dilution Integrity

In several Beagle dog plasma samples, the KTF concentration exceeded 5 ng/mL, which is the ULOQ. Therefore, dilute validation was performed to accurately quantify the concentration of KTF in these samples. Dilution integrity was confirmed by spiking plasma with analyte concentrations double those of the QC standards, followed by dilution with blank plasma to achieve the original QC concentrations. Table 5 illustrates the double dilution validation of KTF in Beagle dog plasma.

### 2.3. Incurred Sample Reanalysis (ISR)

ISR was carried out to validate the analytical method developed. Based on the regulatory guidelines outlined [21,22], suitability is determined by ensuring that over 67% of the reanalyzed samples fall within a deviation of ±20% from the initial analysis value. In this study, out of a total of 288 samples, 29 were randomly selected for reanalysis using the Stata/SE ver. 11.0 program. The results of the reanalysis confirmed that all samples met the acceptance criteria for KTF [23].

### 2.4. Application to a Bioequivalence Study

**Figure 3:**
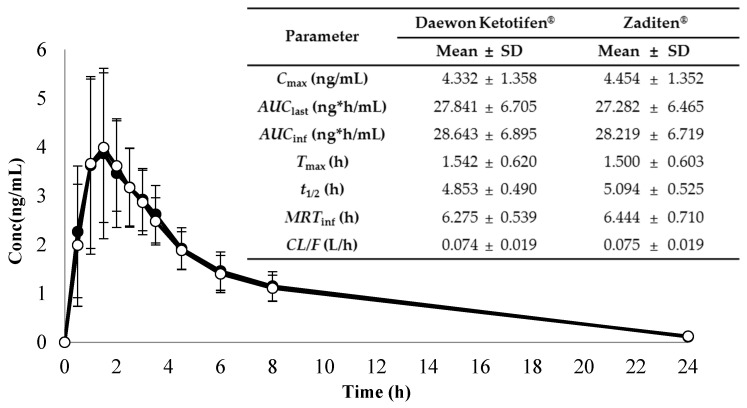
Mean plasma concentration-time curves and pharmacokinetic parameter estimates of 12 Beagle dogs following administration of 2 mg of ketotifen (●: Daewon ketotifen^®^ syrup [Daewon Pharmaceutical Co., Ltd., Seoul, Republic of Korea]; ○: Zaditen^®^ syrup [Novartis Co., Ltd., Seoul, Republic of Korea]) in pharmacokinetics test.

The application of the analytical method involving the API 4000 LC–MS/MS system was effectively executed to assess the biological equivalence of two ketotifen syrup formulations (Daewon ketotifen^®^ syrup and Zaditen^®^ syrup) by analyzing 288 Beagle dog plasma samples. The mean ± standard deviation plasma concentration-time profiles and pharmacokinetic parameters when a dose of 2 mg/day KTF syrup formulation was orally administered to 12 Beagle dogs are shown in Figure 3. Non-compartmental analysis to derive pharmacokinetic parameters was performed using WinNonlin (version 6.3). The *C*_max_ values for Daewon ketotifen^®^ syrup and Zaditen^®^ syrup were 4.332 ± 1.358 and 4.454 ± 1.352 ng/mL, respectively. The *T*_max_ values were 1.542 ± 0.620 and 1.500 ± 0.603 h, respectively, and the *t*_1/2_ values were 4.853 ± 0.490 and 5.094 ± 0.525 h, respectively. Additionally, their *AUC*_last_ values were 27.841 ± 6.705 and 27.282 ± 6.465 ng*h/mL, respectively, and the *AUC*_inf_ values were 28.643 ± 6.895 and 28.219 ± 6.719, respectively. Lastly, their *MRT*_inf_ values were 6.275 ± 0.539 and 6.444 ± 0.710 h, respectively, and the *CL/F* values were 0.074 ± 0.019 and 0.075 ± 0.019 L/h, respectively. According to the ANOVA results of the Beagle dog study, the mean ratio of Daewon ketotifen syrup^®^ to Zaditen syrup formulation for *AUC*_last_ and *C*_max_ was 1.0194 and 0.9681, respectively. The 90% confidence intervals for the ratios of *AUC*_last_ and *C*_max_, which were log-transformed as log 0.9840–log 1.0561 and log 0.8847–log 1.0592, respectively, fell entirely within the regulatory acceptance limits for bioequivalence (80–125%) [24]. Thus, it can be interpreted that Daewon ketotifen^®^ syrup and Zaditen^®^ syrup are biologically equivalent in Beagle dogs. Previous studies have evaluated the pharmacokinetics of orally administered KTF in rabbits and humans [7,9,10,11]. Male Japanese albino rabbits (2.7−3.3 kg) were orally administered KTF at doses of 1 mg/kg and 5 mg/kg via capsule formulation. The plasma concentration of KTF at a dose of 1 mg/kg was mostly below the detection limit. Therefore, the 5 mg/kg dose data were adjusted to reflect a 1 mg/kg dose. The *C*_max_ and *T*_max_ values for the adjusted 1 mg/kg dose were 15.8 ± 2.00 ng/mL and 1.51 ± 0.25 h, respectively [10]. In addition, healthy adults were orally administered a 2 mg dose of KTF in tablet dosage form for Zaditen^®^ and Fumatifen^®^. As a result, the *C*_max_, *T*_max_, and *AUC*_last_ for the Zaditen^®^ tablets were 0.20074 ± 0.09852 ng/mL, 2.77 ± 0.77 h, and 1.926 ± 0.915 ng*h/mL, respectively. The *C*_max_, *T*_max,_ and *AUC*_last_ for Fumatifen^®^ tablets were 0.19991 ± 0.10680 ng/mL, 2.65 ± 0.70 h, and 1.972 ± 1.092 ng*h/mL, respectively. The 90% confidence intervals for the ratios of the area under the curve at 0–36 h (*AUC*_last_) and *C*_max_ were log 0.9241–log 1.0636 and log 0.9165–log 1.0550, respectively, which met the bioequivalence criteria [9,24]. In another study conducted on healthy adults, KTF was administered orally in two dosage forms (capsule and syrup) at a dose of 2 mg for both Hexal^®^-pharma and Zaditen^®^. For pharmacokinetic research, the results showed that the *C*_max_ values for Hexal^®^-pharma and Zaditen^®^ in capsule form were 281 ± 84 and 270 ± 66 pg/mL, respectively, and the *T*_max_ values were 3.9 ± 2.2 and 3.6 ± 1.6 h, respectively. Additionally, the *t*_1/2_ values were 13.1 ± 6.0 and 18.3 ± 6.7 h, and the *AUC*_last_ values were 3008 ± 1556 and 3390 ± 1260 pg*h/mL, respectively. The *AUC*_inf_ values were 4703 ± 2057 and 4941 ± 1813 pg*h/mL, respectively. In syrup form, the *C*_max_ values for Hexal^®^-pharma and Zaditen^®^ were 421 ± 206 and 355 ± 147 pg/mL, respectively, and the *T*_max_ values were 2.9 ± 1.9 and 2.3 ± 1.2 h, respectively. Additionally, the *t*_1/2_ values were 12.2 ± 4.5 and 12.7 ± 5.6 h, and the *AUC*_last_ values were 3984 ± 2359 and 3387 ± 2103 pg*h/mL, respectively. The *AUC*_inf_ values were 4970 ± 2607 and 3815 ± 1513 pg*h/mL, respectively [11]. In previous two human studies at the same dosage, the syrup formulation resulted in a higher *C*_max_ and faster *T*_max_. This suggests that the absorption rate and extent of KTF are faster in the syrup formulation than in the tablet and capsule formulations. However, in our study, a clear difference in the syrup formulation was observed in the pharmacokinetic values of *AUC*_last_. These differences may be attributed to the previous study being conducted in humans, possibly influenced by interspecies differences in the activity of CYP3A4, which is primarily involved in the metabolism of KTF [25]. Although dogs are useful and convenient models for humans, there can be differences in the oral pharmacokinetic profiles of the two species [13]. Therefore, there are many additional factors to consider when obtaining pharmacokinetic data.

In summary, the syrup form exhibited the highest *C*_max_ and fastest *T*_max_ among capsule and tablet formulations. Thus, the syrup form holds pharmacokinetic significance in facilitating the faster and more effective absorption of KTF. Therefore, our study confirmed the biological equivalence of these two syrup formulations, providing valuable information for future research on KTF.

## 3. Materials and Methods

### 3.1. Chemicals and Reagents

Daewon ketotifen^®^ (ketotifen fumarate, purity 100.0%) was obtained from Daewon Pharmaceutical Co. (Seoul, Republic of Korea), and ketotifen-d3 fumarate (IS, purity 99.23%, isotopic 99.7%) was obtained from ChemScene (Monmouth Junction, NJ, USA). The water used in the entire analysis was prepared using tertiary purified water (DW) from an AQUAmax^®^ water purification system (YoungLin Co., Anyang-si, Republic of Korea). HPLC–grade acetonitrile, methanol, and methyl tertiary-butyl ether (MTBE) were purchased from JT Baker (Phillipsburg, NJ, USA). The sodium hydroxide, ammonium formate, and formic acid were obtained from Sigma-Aldrich (Steinheim, Germany). Control Beagle dog plasma with heparin was procured from NDIC (Gwangju-si, Gyeonggi-do, Republic of Korea). NDIC stands for Nonclinical Dictionary (Nonclinical CRO) and is the organization responsible for collecting blood from Beagle dogs.

### 3.2. Liquid Chromatographic Conditions

The Nexera X2 HPLC system (Shimadzu, Tokyo, Japan) was utilized for the separation process. The chromatographic separation was performed using Luna^®^ Hilic column (50 × 2.0 mm i.d., 3 μm; Phenomenex, Torrance, CA, USA) maintained at 45 °C. The other columns used for the tests were Hydrosphere^®^ C18 (50 × 2.1 mm, 3 µm, YMC, Kyoto, Japan), Luna^®^ C18 (50 × 2.0 mm, 3 µm, Phenomenex), and Halo^®^ C18 (100 × 2.1 mm, 2.7 µm, Advanced-Materials Technology, Wilmington, DE, USA). The mobile phase of the experiment comprised two components: solvent A that was adjusted to a pH of 3.0 using formic acid in 10 mmol/L ammonium formate, and solvent B, which contained 0.05% formic acid in acetonitrile. The two solvents were combined in an isocratic ratio of 5:95 (*v*/*v*) and delivered at a flow rate of 0.2 mL/min. The isocratic ratios tested before establishing the conditions were 20:80 (*v*/*v*) and 2:98 (*v*/*v*). The temperature of the autosampler was adjusted to 10 °C, and 7 μL of each prepared sample was injected into the LC–MS/MS system for analysis for 3 min.

### 3.3. Mass Spectrometric Conditions

Mass spectrometry was performed on an API 4000 triple-quadrupole mass spectrometer (AB SCIEX, Framingham, MA, USA) using positive ion mode. The MS/MS conditions were optimized for quantification of both KTF and IS as follows: Gas 1 (GS1) at 275.8 kPa, Gas 2 (GS2) at 413.7 kPa, ion spray voltage (IS) set to 5500.0 V, turbo heater temperature (TEM) maintained at 450.0 °C, interface heater (Ihe) turned ON, entrance potential (EP) set to 10.0 V, collision activation dissociation (CAD) at 48.3 kPa, and curtain gas (CUR) at 124.1 kPa. Additionally, the DP, CE, and CXP of KTF and IS applied are shown in Table 6. The data acquisition and quantification for KTF were performed using Analyst^®^ Classic software version 1.6.2. (AB SCIEX, Framingham, MA, USA).

### 3.4. Preparation of Calibration Standard Solutions and Quality Control Samples

A concentration of 1 mg/mL was prepared in methanol for the standard stock solutions of KTF and IS. The working solutions of various concentrations were prepared by diluting the standard stock solutions with acetonitrile. The concentrations of the working solutions for the calibration curve (CC) were 0.2, 0.5, 1, 5, 10, 20, and 50 ng/mL, and quality control (QC) were 0.2, 0.6, 4, and 40 ng/mL, with an IS concentration of 25 ng/mL. All stock and working solutions were stored at −20 °C until used. The corresponding working solutions prepared were diluted with blank Beagle dog plasma. The calibration standards were calibrated to have final concentrations of 0.02, 0.05, 0.1, 0.5, 1, 2, and 5 ng/mL, while the QC samples had concentrations of 0.02, 0.06, 0.4, and 4 ng/mL.

### 3.5. Plasma Sample Preparation

The Beagle dog plasma samples were removed from a −70 °C deep freezer before pretreatment and thawed at room temperature. Each sample was vortexed and centrifuged to ensure complete mixing of the contents. An aliquot of 300 μL from each sample was transferred to a glass tube, to which 20 μL of IS (25 ng/mL) and 20 μL of 10 mmol/L sodium hydroxide solution were added. Subsequently, 5 mL of extraction solvent, MTBE, was added to the mixture and vortexed for 15 min. The tubes were then centrifuged at 2691× *g* at 4 °C for 10 min and frozen for 10 min in a −70 °C deep freezer. The organic layer from the top (5 mL) was subsequently moved to a separate tube and dried using a N_2_ evaporator at 40 °C under a flow of nitrogen gas.

The dried residue was reconstituted with a mixture of 95% acetonitrile (D.W.: acetonitrile = 5:95, *v*/*v*) and 0.05% formic acid in acetonitrile (50:50, *v*/*v*) to a total volume of 300 μL and then loaded into the autosampler. The LC–MS/MS system received only 7 μL of the reconstituted volume.

### 3.6. Method Validation

The bioanalytical method was validated in compliance with the guidelines issued by the Ministry of Food and Drug Safety in Korea (MFDS) and the U.S. Food and Drug Administration (FDA) [21,22]. Validated analytical methods were verified for selectivity, lower limit of quantitation (LLOQ, 0.02 ng/mL), linearity, carryover, precision, accuracy, recovery, matrix effect, and stability, and following the validation guidelines of the bioanalytical method.

#### 3.6.1. Selectivity and Lower Limit of Quantitation

The presence of endogenous material that could be eluted simultaneously with the analyte and IS in Beagle dog plasma was assessed by analyzing six different lots, which demonstrated selectivity. The aforementioned sample preparation procedure was employed to treat these samples. As a result, no interfering peaks were observed. LLOQ means the lowest plasma concentration of KTF in the Beagle dog plasma. The signal-to-noise (S/N) ratio for all batches exceeded 10, and the deviation from both precision and accuracy was less than 20%.

#### 3.6.2. Linearity and Carryover

In the calibration curve, the linear regression with a weighting of 1/*x*^2^ was used to determine linearity, and the range of KTF was 0.02–5 ng/mL for the Beagle dog plasma. The calibration curves were represented by *y* = *ax* + *b*, with *y* being the mean of the peak area ratios of the analytes to their internal standards, *a* representing the slope of the calibration curves, *b* representing the y-axis intercept of the calibration curve, and *x* standing for the analyte concentration. Acceptance criteria were % accuracy should be within 85 to 115%, except LLOQ; for LLOQ it should be 80 to 120%; and % CV should be ±15% except LLOQ; for LLOQ it should be 20% [26,27]. The carry-over was used to verify that there was no transfer to the next concentration when the highest concentration was injected. This was determined by injecting double-blank samples after injecting ULOQ and LLOQ samples. If the areas of interfering peaks were below 20% of the LLOQ for the analyte and less than 5% of the IS, they were considered acceptable.

#### 3.6.3. Precision and Accuracy

Intra- or inter-day precision and accuracy of the method were determined by 5 duplicate analyses of LLOQ (0.02 ng/mL), low QC (LQC, 0.06 ng/mL), middle QC (MQC, 0.4 ng/mL), and high QC (HQC, 4 ng/mL) either on the same day or on three consecutive days. The average of the accuracy and precision of evaluation criteria was required to be within ±15% deviation from the normal value except LLOQ, which was set at ±20%.

#### 3.6.4. Recovery and Matrix Effect

The evaluation of recovery and matrix effects involved conducting repeated measurements on six samples each at LQC, MQC, and HQC levels, using three distinct sample preparation methods.: pre-extraction with analyte spiking into the matrix ([A]), post-extraction spiking after matrix extraction ([B]), and spiking of pure analyte solutions into the reconstitution solvent ([C]). The recoveries of KTF and IS were calculated as (A/B × 100)%. The matrix effect of KTF and IS was determined as (B/C × 100)%, allowing evaluation of ion enhancement or suppression caused by the plasma matrix.

#### 3.6.5. Stability

The stability of the stock and working solution was assessed by comparing the KTF and IS peak areas of three replicates of low and high QC at room temperature for 3 h and refrigerated conditions (−20 °C) for 7 h with those of freshly prepared solutions. The stability of analytes in Beagle dog plasma was assessed under various conditions: room temperature, refrigerated condition (4 °C), frozen condition (−70 °C) for 7 h, freezing and thawing (3 cycles), autosampler (10 °C) stability for 30 h.

#### 3.6.6. Dilution Validation

Dilution validation was performed due to several Beagle dog plasma samples exceeding the ULOQ concentration. The dilution of these samples was achieved using pooled plasma from Beagle dogs, and they were subsequently reexamined. The QC samples of diluted KTF were prepared at twice the original concentrations of 0.06, 0.4, and 4 ng/mL. After spiking into plasma, the samples were prepared by diluting them two-fold using Beagle dog blank plasma to achieve the original QC concentrations. The acceptance criteria require that the precision and accuracy, as assessed by injecting the QC concentrations five times, fall within 15%.

### 3.7. Application to a Bioequivalence Study

Healthy twelve male Ridglan Beagle dogs weighing 11.73 ± 0.83 kg were purchased from Raonbio Inc. (Yongin-si, Gyeonggi-do, Republic of Korea). Beagle dogs were maintained under standardized laboratory conditions (temperature, 21 ± 2 °C, RH: 35–65%). All of the experiments received approval from the Animal Ethics Committee at NDIC (Gwangju-si, Gyeonggi-do, Republic of Korea, protocol code: L2023065; date of approval: 21 March 2023). This study involved 12 Beagle dogs, which were randomly divided into 2 groups (*n* = 6 per group). A newly developed validated method was employed in a cross-over design to assess the bioequivalence of KTF. The experiment compared two oral medications: Daewon ketotifen^®^ syrup (the test product from Daewon Pharmaceutical Co., Ltd., Republic of Korea) and Zaditen^®^ syrup (the reference product from Novartis Co., Ltd., Republic of Korea). The clinical dose was set at 2 mg per subject. After overnight fasting (16 h), Beagle dogs were administered a single oral dose of 2 mg KTF syrup. Afterward, 1.5 mL of whole blood was drawn using a 3 mL syringe into heparin-containing tubes at the following intervals: 0 (pre-dosing), 0.5, 1, 1.5, 2, 2.5, 3, 3.5, 4.5, 6, 8, and 24 h. Centrifugation was utilized to obtain 700 μL of plasma samples at 3515× *g* for 10 min at 4 °C, followed by transfer to a tube and immediate storage at −70 °C for future analysis.

### 3.8. Incurred Sample Reanalysis

To evaluate the consistency of pharmacokinetic analyses, an Incurred Sample Reanalysis (ISR) was performed. It involves randomly selecting 29 samples, which represent 10% of the total samples, for analysis. The acceptable criterion is that the concentration deviation of the samples, compared with their initial analysis values, should be within ±20%, and at least 67% of the analyzed ISR samples must meet this criterion [23]. Through this analysis, the reproducibility of the pharmacokinetic profile, including *C*_max_ (maximum concentration) and elimination phase, can be determined.

### 3.9. Statistical Analysis of Bioequivalence

Pharmacokinetic parameters such as *C*_max_ (maximum plasma drug concentration), *T*_max_ (time to reach *C*_max_), terminal half-life (*t*_1/2_), *AUC*_inf_ (area under the plasma concentration–time curve from time zero to infinity), *AUC*_last_ (area under the plasma concentration–time curve from time zero to the time of the last measurable concentration), *MRT*_inf_ (mean residence time extrapolated to infinity.), and *CL/F* (apparent systemic clearance) of KTF within each group were extrapolated and calculated using noncompartmental methods (WinNonlin version 8.1; Pharsight Corporation, Mountain View, California). For the purpose of bioequivalence analysis, *AUC*_last_ and *C*_max_ were considered as primary variables. Bioequivalence of two formulations was assessed by means of an analysis of variance (ANOVA) for crossover design and calculating 90% confidence intervals (CIs) of the ratio of test (Daewon ketotifen^®^ syrup)/reference (Zaditen^®^ syrup) using log-transformed data. FDA (Food and Drug Administration) guidelines specify that the criterion for bioequivalence is that the log-transformed values should fall within the range of 0.8 to 1.25 [24].

## 4. Conclusions

In this study, a method for the analysis of KTF concentration in the plasma of Beagle dogs using LC–MS/MS was developed and validated. The developed method was sensitive, reproducible, precise, and accurate. Using the Luna^®^ Hilic column, the elution time of the analyte was sufficiently separated from matrix peaks arising from endogenous substances in Beagle dog plasma. Furthermore, a low LLOQ (0.02 ng/mL) was established while using minimal amounts of Beagle dog plasma (300 μL). The LLE method, employing MTBE as the extraction solvent in sample pretreatment, was employed to reduce matrix effects and increase recovery rates. This approach was successfully employed in pharmacokinetic research involving KTF given orally as a syrup to Beagle dogs. By observing a higher *C*_max_ and earlier *T*_max_ for the syrup formulation studied in our research compared to the known *C*_max_ and *T*_max_ for capsule and tablet formulations in several human studies, it was evident that a more rapid effect could be achieved with the syrup formulation. Owing to the clear differences between humans and Beagle dogs, additional diverse tests would be necessary to verify the bioequivalence of the two formulations based on this study. However, since the bioequivalence between Daewon ketotifen^®^ syrup and Zaditen^®^ syrup has been confirmed, this study appears to be effectively applicable to future research on KTF that will be conducted later.

## Figures and Tables

**Table 1:** Regression analysis of making validation methods of ketotifen in Beagle dog plasma (*n* = 9).

Compound	Number	Linearity			
		Slope	Intercept	*r*	*r* ^2^
Ketotifen	1	0.588	−0.001270	0.9987	0.9974
	2	0.604	−0.002100	0.9995	0.9990
	3	0.584	−0.000593	0.9991	0.9982
	4	0.607	−0.001070	0.9987	0.9974
	5	0.577	−0.001480	0.9981	0.9962
	6	0.557	0.001020	0.9974	0.9948
	7	0.598	−0.002820	0.9988	0.9976
	8	0.559	0.001380	0.9984	0.9968
	9	0.567	−0.000405	0.9977	0.9954

**Table 2:** Intra-day and inter-day precision and accuracy of the LC–MS/MS method for determining ketotifen concentration in Beagle dog plasma.

Compound	Nominal Concentration (ng/mL)	Intra-Day (*n* = 5)		Inter-Day (*n* = 15)		
		Mean ± SD (ng/mL)	Precision (CV, %) ^a^	Mean ± SD (ng/mL)	Precision (CV, %)	Accuracy (%) ^b^
Ketotifen	0.02	0.02 ± 0.00	6.67	0.02 ± 0.00	7.56	101.07
	0.06	0.06 ± 0.00	5.20	0.06 ± 0.00	5.51	97.56
	0.4	0.39 ± 0.01	3.30	0.39 ± 0.01	3.49	97.87
	4	4.25 ± 0.10	2.28	4.25 ± 0.13	3.14	106.31

^a^ CV (%) = (standard deviation of the calculated concentrations/mean concentration) × 100. ^b^ Accuracy (%) = (predicted concentration/nominal concentration) × 100.

**Table 3:** Extraction recovery and matrix factor of ketotifen and ketotifen-d3 from Beagle dog plasma (*n* = 6).

Compounds	Nominal Concentration (ng/mL)	Recovery (%)		Matrix Effect (%)	
		Mean ± SD	Precision (CV)	Mean ± SD	Precision (CV)
**Ketotifen**	0.06	102.30 ± 5.78	5.64	70.01 ± 4.84	6.91
	0.4	99.71 ± 5.00	5.01	67.22 ± 8.73	12.84
	4	100.69 ± 10.27	10.12	73.93 ± 8.40	11.34
**Ketotifen-d3**	25	92.40 ± 8.17	7.94	71.11 ± 6.84	9.58

**Table 4:** Stability data of stock and working solution at room temperature in Beagle dog plasma sample under five different conditions.

Stability Storage Condition	Concentration		
	0.06 (Mean ± SD, %)	0.4 (Mean ± SD, %)	4 (Mean ± SD, %)
Solution stability (%)			
Stock room temperature (3 h)	103.54 ± 5.81		97.70 ± 4.33
Working room temperature (7 h)	96.20 ± 9.22		99.83 ± 6.17
Plasma Sample stability (%)			
Room temperature (7 h)	93.33 ± 0.00	96.25 ± 0.02	102.72 ± 0.12
Refrigeration (7 h, 4 °C)	96.67 ± 0.00	97.42 ± 0.01	100.93 ± 0.05
Freeze-thaw stability (3 cycles)	105.56 ± 0.00	96.25 ± 0.01	103.35 ± 0.08
Autosampler (10 °C, 30 h)	88.33 ± 0.14	97.67 ± 0.01	105.52 ± 0.12
Deep freeze (7 h, −70 °C)	104.44 ± 0.00	96.33 ± 0.00	102.82 ± 0.10

**Table 5:** Results of two-fold dilute validation experiment of ketotifen in Beagle dog plasma (*n* = 5).

Nominal Concentration (ng/mL)	Dilution Factor 2		
	Mean ± SD (ng/mL)	Precision (CV, %)	Accuracy (%)
0.06	0.06 ± 0.00	2.20	98.67
0.4	0.39 ± 0.01	3.40	98.45
4	4.03 ± 0.07	1.73	100.71

**Table 6:** Mass conditions indicated the precursor ion to the product ion of KTF and KTF-d3 (IS).

Compounds	Ion Transition (*m*/*z*)	DP (V)	EP (V)	CE (V)	CXP (V)
KTF	310.2 → 96.0	116.0	10.0	35.0	16.0
KTF-d3	313.2 → 99.1	121.0	10.0	35.0	12.0

DP: declustering potential, EP: entrance potential, CE: collision energy, CXP: cell exit potential.

## Data Availability

The data presented in this study are available upon request.

## Supplementary Materials

The following supporting information can be downloaded at: https://www.mdpi.com/article/10.3390/molecules29184505/s1, Figure S1: Chromatogram of KTF-d3 in solution using Hydrosphere^®^ C18; Figure S2: Chromatograms of KTF and KTF-d3 in plasma using Luna^®^ C18: from top to bottom, double blank plasma (without ketotifen and IS), blank plasma spiked with ketotifen-d3 (IS, 25 ng/mL), blank plasma spiked with ketotifen (LLOQ, 0.02 ng/mL) and ketotifen-d3 (IS, 25 ng/mL); Figure S3: Chromatograms of KTF and KTF-d3 in plasma using Halo^®^ C18: from top to bottom, double blank plasma (without ketotifen and IS), blank plasma spiked with ketotifen-d3 (IS, 25 ng/mL), blank plasma spiked with ketotifen (LLOQ, 0.02 ng/mL) and ketotifen-d3 (IS, 25 ng/mL).
