# Supplementary material for: Development of a Simple and Validated LC–MS/MS Method for Quantitative Determination of Ketotifen in Beagle Dog Plasma and Its Application to Bioequivalence Study of Ketotifen Syrup Dosage Form
Source: Molecules. 2024 Sep 23;29(18):4505. doi: 10.3390/molecules29184505 (PMC11433888; doi:10.3390/molecules29184505)
Supplement: Supplementary file 1 [file molecules-29-04505-s001.zip › molecules-3200867-supplementary.pdf]

Supplementary Materials

Development of a Simple and Validated LC–MS/MS Method for Quantitative Determination of Ketotifen in Beagle Dog Plasma and Its Application to Bioequivalence Study of Ketotifen Syrup Dosage Form

Eunseo Song [1,2,3], Wang-Seob Shim [2], Doowon Choi [1,2,3], Yuna Song [1,2,3], Hyeong Geun Jo [4], Soobok Lee [4,5], Suk Han Jung [4,6], Yeo Jin Choi [3,*] and Kyung-Tae Lee [1,2,3,*]

1 Department of Biomedical and Pharmaceutical Science, Graduated School, Kyung Hee University, Seoul 02447, Republic of Korea; sssk2303@khu.ac.kr (E.S.); atnoon@khu.ac.kr (D.C.); songyu0819@khu.ac.kr (Y.S.)
2 Kyung Hee Drug Analysis Center, College of Pharmacy, Kyung Hee University, Seoul 02447, Republic of Korea; wsshimm@khu.ac.kr
3 Department of Pharmacy, College of Pharmacy, Kyung Hee University, Seoul 02447, Republic of Korea
4 Daewon Pharm. Co., Ltd., 386 Cheonhodae-ro, Gwangjin-gu, Seoul 04808, Republic of Korea; jhgeun@daewonpharm.com (H.G.J.); sblee@daewonpharm.com (S.L.); kshjung@daewonpharm.com (S.H.J.)
5 Department of Regulatory Science, Graduated school, Kyung Hee University, Seoul 02447, Republic of Korea
6 Department of Fundamental Pharmaceutical Science, Graduated School, Kyung Hee University, Seoul 02447, Republic of Korea
* Correspondence: yeojin.choi@khu.ac.kr (Y.J.C.); ktlee@khu.ac.kr (K.-T.L.)

Supplementary data

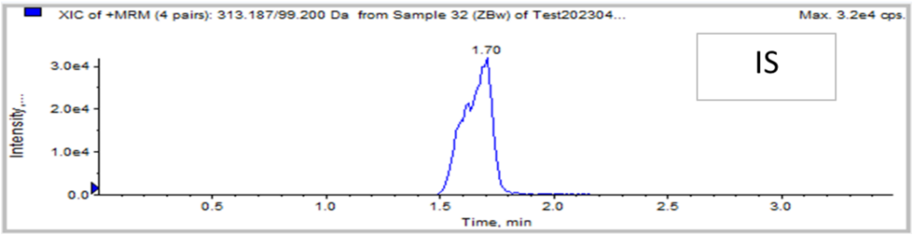

Figure S1. Chromatogram of KTF-d3 in solution using Hydrosphere® C18.

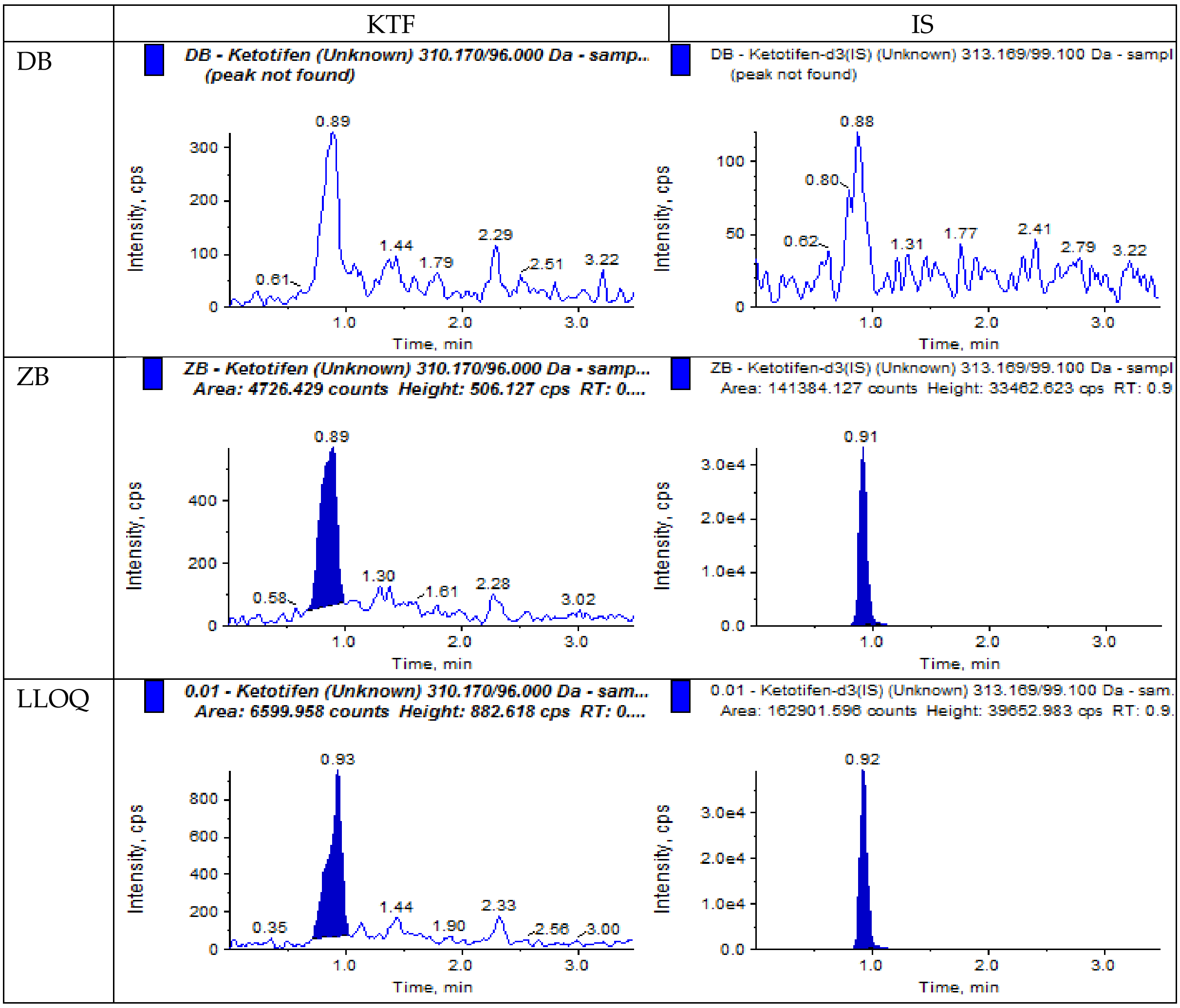

Figure S2. Chromatograms of KTF and KTF-d3 in plasma using Luna® C18: from top to bottom, double blank plasma (without ketotifen and IS), blank plasma spiked with ketotifen-d3 (IS, 25 ng/mL), blank plasma spiked with ketotifen (LLOQ, 0.02 ng/mL) and ketotifen-d3 (IS, 25 ng/mL).

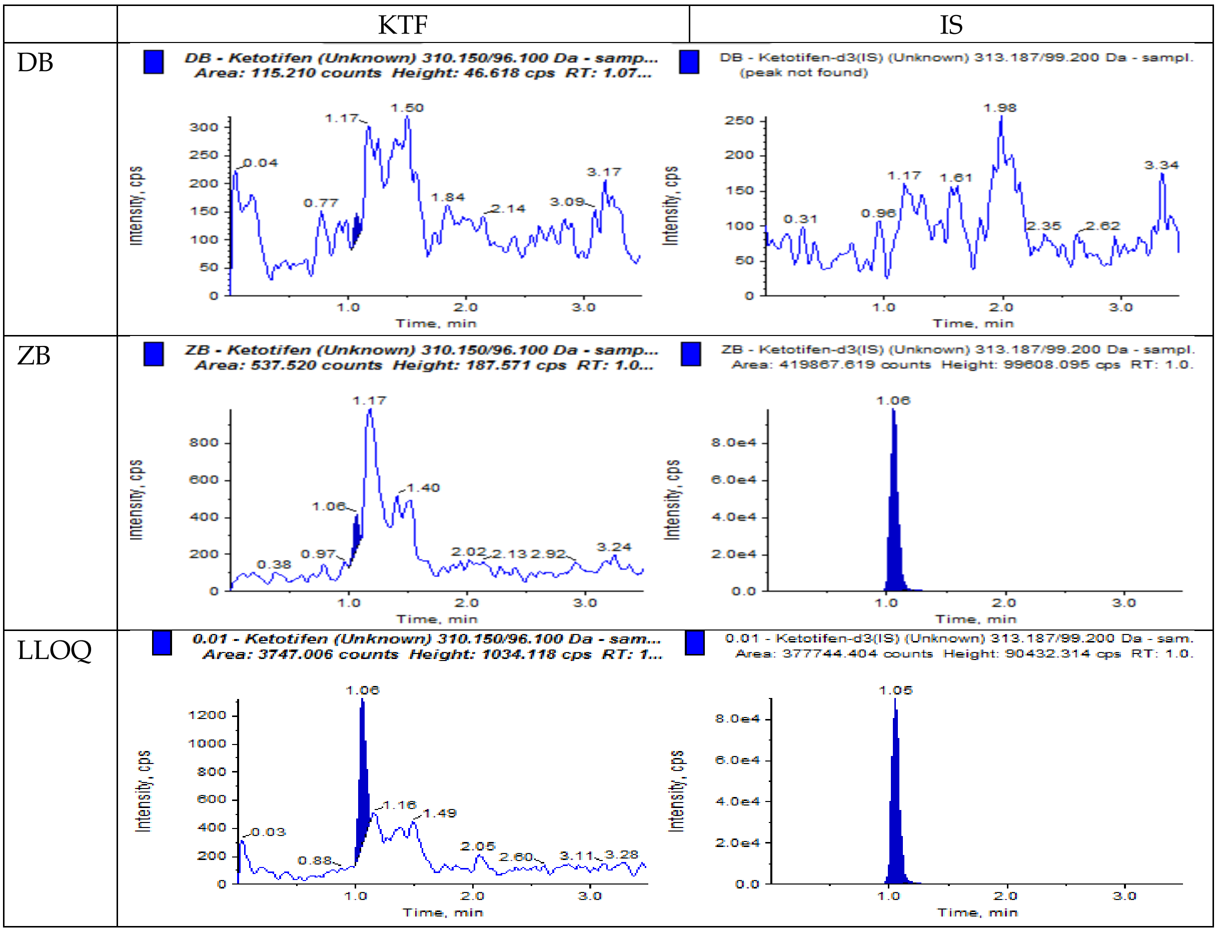

Figure S3. Chromatograms of KTF and KTF-d3 in plasma using Halo® C18: from top to bottom, double blank plasma (without ketotifen and IS), blank plasma spiked with ketotifen-d3 (IS, 25 ng/mL), blank plasma spiked with ketotifen (LLOQ, 0.02 ng/mL) and ketotifen-d3 (IS, 25 ng/mL).
